# Mapping Autoantibodies in Children With Acute Rheumatic Fever

**DOI:** 10.3389/fimmu.2021.702877

**Published:** 2021-07-15

**Authors:** Reuben McGregor, Mei Lin Tay, Lauren H. Carlton, Paulina Hanson-Manful, Jeremy M. Raynes, Wasan O. Forsyth, Diane T. Brewster, Martin J. Middleditch, Julie Bennett, William John Martin, Nigel Wilson, Polly Atatoa Carr, Michael G. Baker, Nicole J. Moreland

**Affiliations:** ^1^ School of Medical Sciences, The University of Auckland, Auckland, New Zealand; ^2^ Maurice Wilkins Centre, The University of Auckland, Auckland, New Zealand; ^3^ The Plant and Food Research Institute, Auckland, New Zealand; ^4^ School of Biological Sciences, The University of Auckland, Auckland, New Zealand; ^5^ Department of Public Health, University of Otago, Wellington, New Zealand; ^6^ Science for Technological Innovation Science Challenge, Callaghan Innovation, Wellington, New Zealand; ^7^ Starship Children’s Hospital, Auckland, New Zealand; ^8^ Waikato District Health Board and Waikato University, Hamilton, New Zealand

**Keywords:** autoantibody, rheumatic fever, protein array, immunoassay, autoantigen, streptococcus A, group A Streptococcus

## Abstract

**Background:**

Acute rheumatic fever (ARF) is a serious sequela of Group A *Streptococcus* (GAS) infection associated with significant global mortality. Pathogenesis remains poorly understood, with the current prevailing hypothesis based on molecular mimicry and the notion that antibodies generated in response to GAS infection cross-react with cardiac proteins such as myosin. Contemporary investigations of the broader autoantibody response in ARF are needed to both inform pathogenesis models and identify new biomarkers for the disease.

**Methods:**

This study has utilised a multi-platform approach to profile circulating autoantibodies in ARF. Sera from patients with ARF, matched healthy controls and patients with uncomplicated GAS pharyngitis were initially analysed for autoreactivity using high content protein arrays (Protoarray, 9000 autoantigens), and further explored using a second protein array platform (HuProt Array, 16,000 autoantigens) and 2-D gel electrophoresis of heart tissue combined with mass spectrometry. Selected autoantigens were orthogonally validated using conventional immunoassays with sera from an ARF case-control study (n=79 cases and n=89 matched healthy controls) and a related study of GAS pharyngitis (n=39) conducted in New Zealand.

**Results:**

Global analysis of the protein array data showed an increase in total autoantigen reactivity in ARF patients compared with controls, as well as marked heterogeneity in the autoantibody profiles between ARF patients. Autoantigens previously implicated in ARF pathogenesis, such as myosin and collagens were detected, as were novel candidates. Disease pathway analysis revealed several autoantigens within pathways linked to arthritic and myocardial disease. Orthogonal validation of three novel autoantigens (PTPN2, DMD and ANXA6) showed significant elevation of serum antibodies in ARF (p < 0.05), and further highlighted heterogeneity with patients reactive to different combinations of the three antigens.

**Conclusions:**

The broad yet heterogenous elevation of autoantibodies observed suggests epitope spreading, and an expansion of the autoantibody repertoire, likely plays a key role in ARF pathogenesis and disease progression. Multiple autoantigens may be needed as diagnostic biomarkers to capture this heterogeneity.

## Introduction

Acute rheumatic fever (ARF) is a serious multi-focal autoimmune sequela of Group A *Streptococcal* (GAS) infection, presenting with a combination of signs and symptoms including one or more of the major manifestations used for diagnosis as part of the Jones criteria (1, 2); arthritis, carditis, Sydenham’s chorea, erythema marginatum and subcutaneous nodules. Approximately 60% of ARF cases progress to chronic rheumatic heart disease (RHD), which can cause permanent heart valve damage (3), with an estimated 33 million people living with RHD globally (4). Although ARF rates declined over the twentieth century, the disease persists in low-income countries and amongst disadvantaged communities in some high-income countries, with Indigenous Māori and Pacific children in New Zealand and Aboriginal children in Australia having some of the highest incidences in the world (5, 6).

The clinical manifestations of ARF usually develop 2-4 weeks after a GAS pharyngitis infection, with growing evidence also implicating GAS skin infections in disease (7). The pathogenesis pathway for ARF remains poorly understood. The current hypothesis involves “molecular mimicry”, wherein antibodies initially targeting specific GAS components are proposed to cross-react with human tissue (8, 9). This is largely based on M-protein specific antibodies and T-cells which cross-react with cardiac myosin, laminin and tropomyosin antigens found in the heart and synovium (9). The role of molecular mimicry remains the subject of debate, with an alternative hypothesis suggesting that infection with GAS causes disruption of the extracellular matrix, which exposes cryptic collagen epitopes, and triggers an autoimmune response (10, 11). Additional autoantibodies could then be generated due to increased inflammation, subsequent tissue damage and epitope spreading (12).

There are few contemporary studies investigating the broader autoantibody response in ARF and a lack of application of unbiased approaches to study the ARF autoantibody repertoire. Protein−microarray technologies enable quantification of autoantibody responses to large proportions of the human proteome (13). These technologies have been used to identify novel autoantibodies and associated disease pathways in a broad range of immune-mediated diseases, including lupus, some cancers and the recently described Multisystem Inflammatory Syndrome in Children (MIS-C) that can develop following SARS-CoV-2 infection (14–16). This study aimed to apply high-content protein-microarray technology to ARF to enable a comprehensive analysis of the disease’s autoantibody landscape. This unbiased array-based approach was taken to inform antibody-driven pathogenesis and identify possible novel disease biomarkers.

## Materials And Methods

### Protein Microarrays

Human Protorrays (Protein microarray platform v5.0) were performed following the manufacturer’s instructions to detect serum autoantibodies (ThermoFisher, Massachusetts, USA). Samples were diluted 1:500 and antibody binding detected with an Alexa Fluor 647 labelled goat anti-human IgG antibody. Arrays were scanned using a GenePix4000B microarray scanner and array grids aligned using the GenePix Pro 5.0 software (Molecular Devices). Raw data were background corrected using the “saddle” correction (17) from the Bioconductor limma package (18), and data were quantile normalized followed by differential expression statistical analysis using linear models and empirical Bayes statistics with the limma package. Proteins antigens with p < 0.05 and a fold-change of > 2.0 were considered significant. For proteins with duplicated identifiers, (proteins with more than one variant on the arrays) variants with the highest absolute fold-change were kept for further analysis. Autoantigens were cross-validated using HuProt v3.0 arrays conducted by CDI Laboratories (Baltimore, USA). Serum antibodies were detected using an Alexa Fluor 532-labelled anti-human IgG secondary and data were quantile normalized as previously described for HuProt arrays (19). Proteins with p < 0.1 and fold−change >1.5 were considered significant.

### Array Analysis and Visualizations

Analysis and visualizations were carried out in R (version 4.0.2) within R studio^19^ (version 1.2.5042) using the tidyverse suite of packages (20). Upset plots were produced using ComplexHeatmap package (21). Venn diagrams were produced using jvenn (22). Heatmaps and hierarchical clustering (using the average Euclidean distance method) were carried out using Morpheus (https://software.broadinstitute.org/morpheus). Disease pathway analysis of differentially bound proteins was carried out using Metascape using custom analysis for enrichment in DisGeNet disease pathways (23, 24). Tissue specificity of proteins was elucidated using “Normal tissue data” downloaded from the human tissue atlas (HPA) from the URL (https://www.proteinatlas.org/about/download) (25). Data were filtered from the HPA using both the “reliability score” and “level”. The Compartments database was also used for filtering proteins *via* the “confidence score” (26). Filtering parameters applied were; an enhanced or supported “reliability score” and high or medium expression “level” in heart muscle, as well as a “confidence score” of >4 for plasma membrane expression or extracellular space.

### Enzyme-Linked Immunosorbent Assays and Interpretation

Selected antigens identified were orthogonally validated in a larger cohort of serum samples using ELISA **(** **Supplementary Table 1**). Antigens were obtained commercially: NM_080423 (protein tyrosine phosphatase non-receptor type 2, PTPN2) (Origene, Maryland, USA), NM_004021 (dystrophin - dp140 variant, DMD) (Origene, Maryland, USA), NM_001155.5 (Annexin VI, ANXA6), (R&D Systems, Minneapolis, USA). For ELISA, Nunc-immunoplates (Sigma-Aldrich, Missouri, USA) were coated with antigen at 2.5 µg/ml (ANXA6) or 2 µg/ml (PTPN2 and DMD) at 4°C overnight and blocked with Phosphate Buffered Saline (PBS) supplemented with 0.5% human serum albumin (Albinorm, Octapharma, Stockholm, Sweden) for 1 hour at 37°C. Following three washes with PBS/0.1% Tween-20, serum was added at a 1:200 dilution in PBS/0.1% human serum albumin for 1 hour at 37°C. IgG binding was detected using goat anti-human IgG labelled with horse-radish peroxidase (Abcam, Cambridge, UK) diluted 1:10,000 in PBS/0.1% human serum albumin, developed with 3,3′,5,5′-Tetramethylbenzidine (TMB) and stopped with 1M HCl. The optical density (OD) at 450 nm was measured using an EnSight absorbance reader. Two serum samples with high and low OD readings for each antigen were included on every ELISA plate as internal positive and negative controls. A CV of <15% between assay runs was set as acceptance criteria.

To compare the individual antigens, and combination of antigens, for performance in discriminating ARF from control groups, the receiver operating characteristic curve (ROC) and area under the curve (AUC) values (from a logistic regression model) for each antigen or combination of all three antigens were calculated by comparing ARF and control samples using the pROC package (27). Confidence intervals for AUC and differences in AUC were obtained using bootstrapping (n=2000) implemented in the pROC package.

### Study Participants

Human sera were obtained from several studies conducted in New Zealand. Each had appropriate ethical approval, and all participants (or their proxies) provided written informed consent. All ARF cases were diagnosed according to the New Zealand modification of the Jones criteria (1, 28). The sera for ProtoArrays were from a study conducted in the Waikato District Health Board region (2012 to 2015; ethics CEN/12/06/017) including ARF (n=3), GAS pharyngitis (n=3), as well as ethnically matched healthy controls (n=3) from the Auckland arm of the children of SCOPE study (29). The sera for the HuProt arrays (ARF with carditis (n=7), ARF without carditis (n=5) and matched healthy controls (n=6)) and ELISA orthogonal validation (ARF n=79 and controls n=85) were from participants recruited as part of the Rheumatic Fever Risk Factors (RF RISK) study (30). This nationwide study conducted between 2014 and 2017 (ethics 14/NTA/53) included first-episode ARF patients and closely matched healthy controls (30). Controls were matched by age, ethnic identification, socioeconomic deprivation (using the New Zealand Deprivation Index score (31)) and geographic area. Sera from children with GAS positive pharyngitis (n=39) used for orthogonal validation ELISAs were recruited as part of a paediatric study investigating GAS skin and throat infections conducted in the Auckland region (2018-2019; ethics 17/NTA/262) (32).

## Results

### Global Analysis of Autoantibody Reactivity in Acute Rheumatic Fever

**Figure 1:**
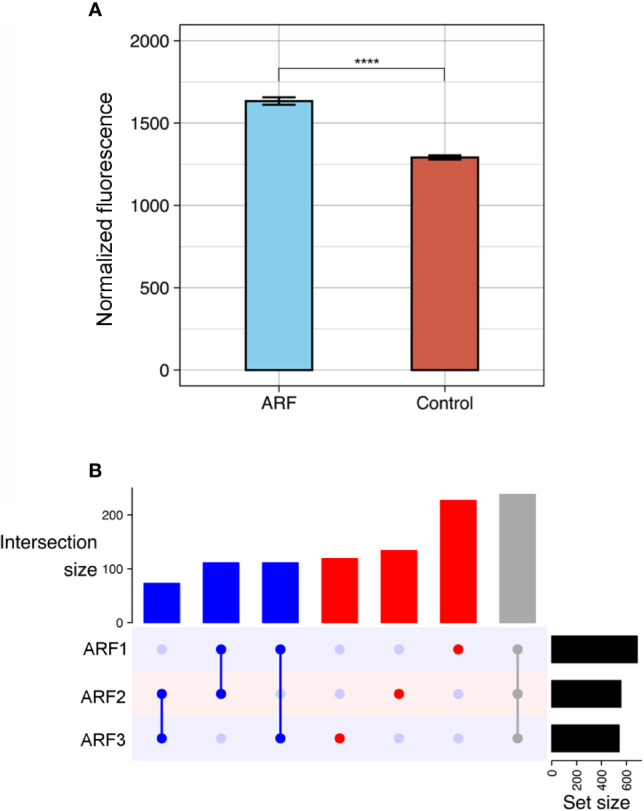
Global autoantibody analysis in ARF using ProtoArrays. **(A)** Overall antibody binding intensities against human proteins in ARF patients (blue, n=3) and grouped controls (red, n=6). Controls comprise both healthy children (n=3) and children with GAS positive pharyngitis (n=3). Bars indicate mean and standard error. ****p < 0.0001 using two sample Wilcoxon test. **(B)** Upset plot showing number of shared autoantibodies between ARF patients (ARF1, 2 and 3). Autoantibodies shared between all three ARF patients are indicated in grey, those shared between at least two patients in blue and those unique to an individual patient in red. The number of autoantibodies in each category is indicated on vertical bar charts with respective colours. Upset plots include only antibodies that showed at least a two-fold enrichment when compared with the mean of grouped controls. Total number of auto-antibodies identified using this threshold per patient is indicated on horizontal bar charts (Set size).

The ProtoArrays initially utilized to profile the autoantibody response in ARF contain over 9000 human proteins expressed in insect cells. As autoantibodies are present in all individuals (33–35), serum binding from ARF patients (n=3) was compared to that of healthy children (n=3) and children with GAS positive pharyngitis (n=3) as controls. Following array QC and normalization, the total antibody reactivity or fluorescence intensity, was determined for each array. ARF arrays showed an increased number of total reactivities compared to controls (p < 0.0001) (**Figure 1A**), suggesting an overall increase in autoantibodies in ARF patient sera. The total reactivity observed on the ARF arrays was markedly increased compared to both the GAS positive pharyngitis and healthy controls (**Supplementary Figure 1**), and as the overarching goal was to identify ARF specific autoantibodies rather than those associated with GAS pharyngitis, the control groups were combined for the subsequent data analysis. The antibody reactivity signals for ARF patients were filtered to include only proteins with a > 2.0 fold-increase in fluorescence intensity compared to the mean of the combined controls (healthy and GAS pharyngitis). This selected for autoantibodies with stronger reactivity in ARF and enabled individual ARF patient’s autoantibody profile to be compared. A total of 1013 autoantibodies showed > 2.0 fold-increased reactivity in ARF compared to the combined controls, with each of the ARF patients having similar numbers of proteins with an increased signal (687 in patient A1, 556 in patient A2 and 541 in patient A3) (**Figure 1B**). Nearly half (47%, 480/1013) of the autoantibodies were unique to an individual patient, with only 23% (238/1013) shared between all three ARF patients and the remaining 29% (295/1013) present in two ARF patients but not the other. Taken together, these results show a global increase in autoantibodies in ARF sera with marked heterogeneity in the autoantibody profiles for each of the ARF patients assessed.

### Autoantibodies Target Proteins in Relevant Disease Pathways

**Figure 2:**
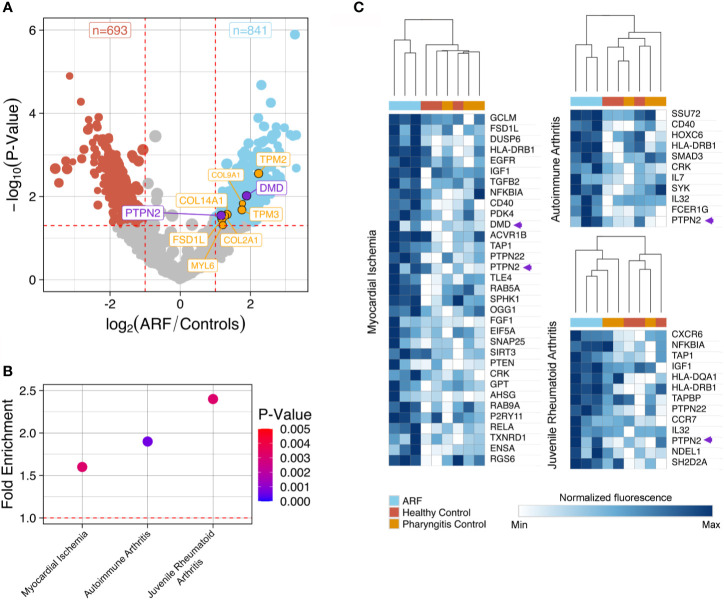
Autoantibody disease pathway analysis using ProtoArray data. **(A)** Volcano plot showing fold-change differences in autoantibody signals between ARF patients (n=3) and controls (n=6). Controls comprise both healthy children (n=3) and children with GAS positive pharyngitis (n=3). The size of the dots and annotations relates to the fluorescence intensity of individual autoantibodies. Red dashed lines indicate cut-offs for significant differences (p < 0.05, fold-change >2). Blue dots represent autoantibodies showing significantly increased binding in ARF patients compared to controls whilst red dots represent autoantibodies showing significantly increased binding in controls compared to ARF patients. Orange annotated autoantibodies have historically been implicated in the pathogenesis of ARF and/or RHD. Purple annotated autoantibodies are novel and of interest for downstream analysis (see **Figure 3**). **(B)** Disease pathway analysis conducted on 841 autoantigens with significantly increased binding in ARF patient sera in part (A). Three significant (p < 0.005) disease pathways are shown in relation to fold enrichment of proteins in pathways (compared to what would be expected by chance). Dot color intensity corresponds to p-value and dashed red line indicates a fold-enrichment of 1, which would represent no enrichment. **(C)** Heat-maps showing individual autoantibody reactivities to proteins belonging to disease pathways identified in part (B). Color intensity corresponds to log_2_ normalized fluorescence values from ProtoArrays of each individual ARF patient (blue columns), healthy controls (red columns) and children with GAS positive pharyngitis (orange columns). A relative color scheme was applied using the min and max values in each row to plot relative colors. The dendrogram represents results of hierarchical clustering on columns. Autoantibody reactivities indicated by purple arrows relate to novel autoantibodies of interest for downstream analysis annotated.

To identify differentially bound proteins in sera from ARF patients and perform pathway analysis, proteins with significantly elevated fluorescence intensity in the ARF group compared with the combined control group were selected (p < 0.05 and fold-change > 2.0). In total, 841 proteins were bound significantly more by ARF serum IgG compared to 693 proteins in controls (**Figure 2A** and **Supplementary Data**). This is in line with the prior global analysis suggesting higher overall autoantibodies in the ARF group. Encouragingly, some proteins that have previously been implicated in the pathogenesis of ARF and RHD were identified (10). These included extracellular matrix proteins; fibronectin (36) and collagens (10, 37, 38) (FSD1L, COL-/2/9/14-A1) as well as intracellular proteins involved in muscle contraction; tropomyosins and myosin (39, 40) (TPM2/3 and MYL6) (**Figure 2A** **)**. As the tropomyosin that previously linked to ARF is cardiac tropomyosin (TPM1), a sequence alignment was performed with the TMP2 and TMP3 isoforms identified in this study. This showed high sequence identity between TPM1 and TPM2 (85.563%) and TPM3 (91.197%) (**Supplementary Figure 2**).

To explore disease connections, the 841 proteins bound by ARF IgG were subjected to disease pathway analysis. This identified three noteworthy pathways, which were both significantly enriched (p < 0.01, fold-enrichment > 1.5) (**Figure 2B** and **Supplementary Data**), and related to two of the major criteria used to diagnose ARF (carditis and arthritis). The “Myocardial Ischemia”, “Autoimmune arthritis” and “Juvenile Rheumatoid Arthritis” disease pathways contain 33, 11 and 13 proteins targeted by autoantibodies in ARF sera, respectively. Unsupervised hierarchical clustering on these pathway proteins shows serum from ARF patients clustering separately from the control groups with respect to fluorescence intensity (**Figure 2C**). This indicates that ARF autoantibodies target a diverse range of proteins that are enriched for in relevant disease pathways.

### Multi-Platform Validation of Array Hits

**Figure 3:**
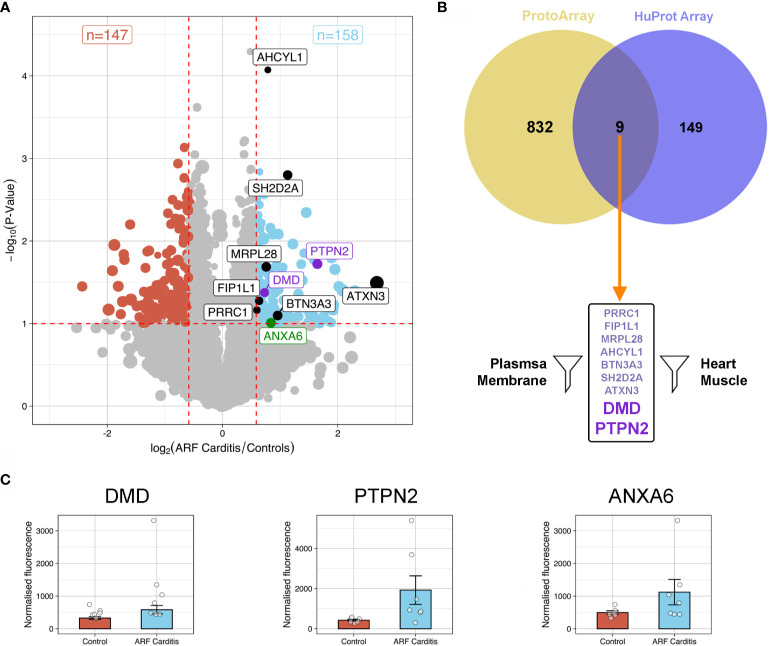
Autoantibody cross-validation using HuProt array **(A)** Volcano plot showing fold-change differences in autoantibody signals between ARF patients with carditis (n=7) and healthy controls (n=6). The size of the dots relates to the fluorescence intensity of individual autoantibodies. Red dashed lines indicate cut-offs for significant differences (p < 0.1, fold-change >1.5). Blue dots represent autoantibodies showing significantly increased binding in ARF patients compared to controls whilst red dots represent autoantibodies showing significantly increased binding in controls compared to ARF patients. Black and purple annotated autoantibodies are those also identified in ProtoArray analysis (see **Figure 2**), with purple dots relating to novel autoantibodies of interest for downstream analysis. Green annotated dot was also identified by 2-DE gel. **(B)** Venn diagram showing the nine overlapping autoantibodies between; 158 proteins identified in HuProt analysis from part (A) in violet; and 841 autoantibodies identified in ProtoArray analysis from **Figure 2A** in yellow. Following filtering for proteins localized to plasma membrane and present in heart muscle, autoantibodies targeting two proteins were identified indicated by large purple text. **(C)** Bar graphs showing mean and standard error of normalized fluorescence values representing autoantibody reactivities to DMD (left), PTPN2 (middle) and ANXA6 (right), from healthy controls (blue, n=6) and ARF patients with carditis (red, n=7).

To further explore and validate the ARF autoantibody repertoire, including antigens identified *via* the ProtoArray analysis, additional high-content arrays (HuProt arrays, > 16,000 human proteins, expressed in yeast cells) were conducted using a distinct cohort of patients; ARF patients with carditis (n=7), ARF patients without carditis (n=5) and healthy controls (n=6). A focussed analysis of the HuProt array data identified 158 human proteins bound significantly more by serum IgG in carditis patients compared to healthy controls (p < 0.1 and fold change >1.5) (**Figure 3A**). Interestingly, one of these proteins, ANXA6, was previously identified in a pilot mass spectrometry analysis of 2−Dimensional Electrophoresis (2-DE) separated human heart lysate and ARF sera conducted in our laboratory (**Supplementary Methods**). Nine of the 158 proteins identified *via* the HuProt analysis overlapped with those identified by the ProtoArrays (**Figure 3B**). When these nine proteins were filtered for expression in heart muscle [using human protein atlas data (25)] as well as localization in or near the plasma membrane [using the compartments database (26)] just two proteins remained; PTPN2 and DMD (**Figure 3B** and **Supplementary Data**). When the same analysis and filtering was applied to the control groups, five overlapping proteins were identified, but none of these passed the filters for expression location. Plotting normalized fluorescence values from the HuProt arrays for PTPN2 and DMD plus ANXA6 illustrates the increased autoantibodies in ARF compared to healthy controls (**Figure 3C**).

### Orthogonal Validation Using ELISA

**Figure 4:**
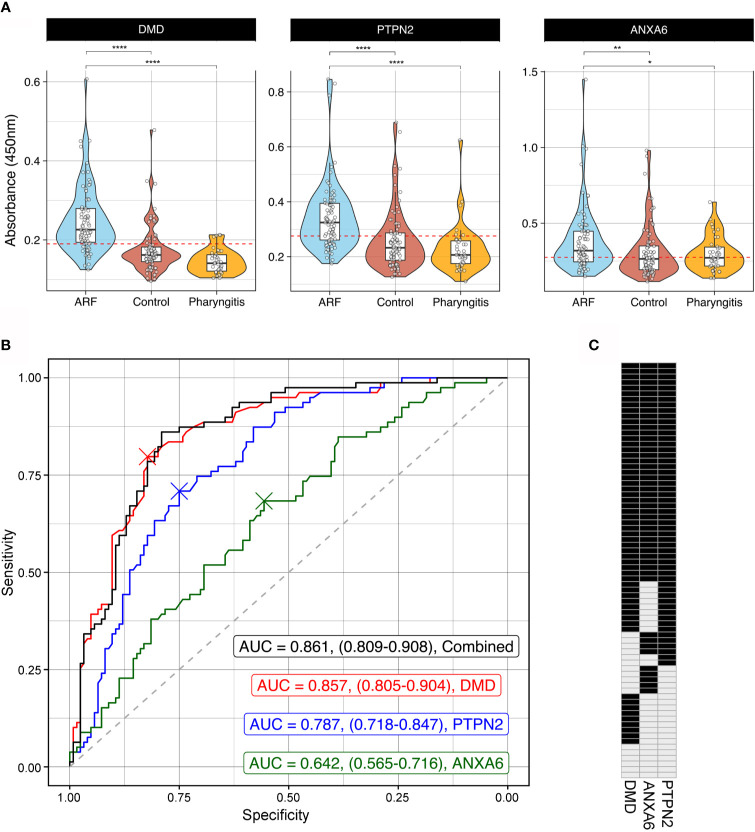
Orthogonal validation of autoantigens by ELISA **(A)** Combined violin and box and whisker plots showing ELISAs targeting DMD (left), PTPN2 (middle) and ANXA6 (right) using sera from ARF patients (blue, n=79), matched healthy controls (red, n=85) and children with GAS positive pharyngitis (orange, n=39). For box and whisker plots the lower and upper hinges correspond to the first and third quartiles (the 25th and 75th percentiles). The whiskers extend from the hinge to the largest and smallest value no further than 1.5 x inter-quartile range from the respective hinge. The violin plot extends from the highest to the lowest value showing density of data. Red dashed line represents the cut-off for positivity positive based analysis in part (B). *p < 0.05, **p < 0.01, ****< 0.0001 using two sample Wilcoxon test. **(B)** Receiver Operator Curves (ROC) of ELISA results from DMD (red), PTPN2 (blue) and ANXA6 (green) as well as all three antigens combined (black). Grey dashed line represents the line of no discrimination, which would indicate a test with no predictive power. The AUC for each analysis is indicated with confidence intervals obtained using bootstrapping in brackets. The crosses represent the optimal cut-off for each autoantigen ELISA. **(C)** Barcode of all 79 ARF patients indicating positive (black) or negative (grey) reactivity to all three autoantigens. Cut-off for positivity was determined from the ROC analysis in part (B) and is represented visually as a red dashed line in part (A).

To orthogonally validate hits from the arrays, ELISAs were performed with DMD, PTPN2 and ANXA6 as antigens. These antigens represent different aspects of ARF disease mechanisms including an immune cell signalling protein [PTPN2 (41)], a central component of the extracellular matrix in muscle fibre [DMD (42)] and a protein abundantly expressed in cardiomyocytes and chondrocytes during osteoarthritis [ANXA6 (43, 44)]. A large cohort was used for validation comprising sera from children with first-episode ARF (n=79), closely matched healthy controls (n=85), as well as children with GAS pharyngitis (GAS positive throat swab and elevated streptococcal serology, n=39) (**Supplementary Table 1**). Significantly elevated autoantibodies were observed in the ARF patient group compared to both healthy controls and the GAS pharyngitis group for all three antigens (**Figure 4A**). The lack of reactivity to these antigens in the GAS pharyngitis group confirms that these autoantibodies are associated with disease, and not with the prior GAS infection. Receiver Operator Curves (ROC) were generated to assess the predictive performance of each of the three antigens alone as well as in combination to distinguish ARF sera from the combined controls (healthy and GAS pharyngitis) (**Figure 4B**). The area under the curve (AUC) metric showed DMD had the best predictive performance (AUC = 0.857, CI:0.805-0.904 followed by PTPN2 (AUC = 0.787, CI:0.718-0.847) and ANXA6 (AUC = 0.642, CI:0.565-0.716). DMD performed significantly better than PTPN2 (p < 0.05) which, in turn, performed significantly better than ANXA6 (p < 0.001). There was no significant gain in performance, above DMD alone, when combining all three assays (AUC = 0.861, CI:0.809-0.908, p = 0.583).

To further explore the biomarker potential of these three antigens, cut-offs for positivity were determined using the Youden index (45). This method identifies an optimal cut-off from the ROC curve that maximizes sensitivity and specificity and resulting values were DMD = 0.190, PTPN2 = 0.274 and ANXA6 = 0.275 (**Figure 4A**). These cut-offs were applied and the ARF patients were categorized as having positive or negative reactivity to each antigen in the form of an autoantibody barcode (**Figure 4C**). In keeping with the superior predictive performance of DMD in the ROC analysis, this antigen yielded the highest number of positive ARF cases (62/79, 78%). However, the barcode also illustrates that patients with first episode ARF have every combination of biomarkers tested ranging from positive for one, two or three antigens through to negative for all three antigens. This further highlights the heterogeneity of autoantibody responses in ARF.

## Discussion

This study has comprehensively investigated the serum autoantibody repertoire in ARF patients using multiple approaches. High content arrays revealed an overall increase in autoantibodies in ARF, with a large proportion of the antibodies unique to individual patients. The current pathogenesis models for ARF following GAS infection are centred on molecular mimicry and the development of autoantibodies to host coiled-coil proteins and extracellular matrix disruption and exposure of cryptic epitopes (10, 11). While the array analysis in this study did identify autoantibodies to myosin, tropomyosin and collagens that might support mimicry and extracellular matrix disruption, the breadth of the autoantibody reactivity observed also points to epitope spreading playing a role in pathogenesis (12).

Epitope spreading, that is the involvement of antigens beyond those that initially trigger the autoimmune response, is thought to be central to the pathogenesis of other systemic autoimmune diseases such as rheumatoid arthritis (46). It has also been suggested to have a role in ARF pathogenesis (12), and is in keeping with the systemic and heterogeneous nature of symptoms associated with the disease including poly-migratory arthritis, carditis, subcutaneous nodules and in some, neurological symptoms or chorea (1, 8). The pathway analysis applied to the array data in this study enabled filtering of the large number of autoantibodies identified and revealed antigens associated with disease pathways relevant to ARF symptoms; “Myocardial Ischemia”, “Autoimmune Arthritis” and “Juvenile Rheumatoid Arthritis”. Yet even within these pathways a diverse range of antigens were targeted by patient serum antibodies, suggesting that a loss of tolerance and epitope spreading may occur in relevant tissues. This is consistent with an ARF pathogenesis model in which a loss of tolerance, driven by inflammation, enhances a dysregulated immune response. There is increasing evidence to suggest that repeated GAS infections prime the immune response for a loss of tolerance in ARF (47–49), and it follows that the presence of inflammation observed in ARF patients (12, 50) could enhance the dysregulated autoimmune response, counteracting tolerance mechanisms, and contribute to epitope spreading and further damage in specific tissues.

In order to validate the analysis of the high content protein arrays, the presence of autoantibodies to three of the antigens identified, DMD, PTPN2 and ANXA6, was assessed in a large ARF cohort. While there was a significant increase in autoantibodies to each of these three antigens in ARF, there was also variability at an individual patient level such that a continuum of reactivity was observed, ranging from autoantibodies to all three antigens in some patients to an absence of autoantibodies to the three antigens in others. The three proteins validated in this study were selected based on their detection across multiple analyses and represent different aspects of potential pathways and tissues involved in ARF. In particular immune signalling [PTPN2 (41)], cardiac tissue [ANXA6 (43), DMD (42)] and joint tissue [ANXA6 (44)]. However, all appear to be associated with the plasma membrane rather than being fully extracellular antigens. It is therefore possible that these antigens are not involved in initiating disease, but rather are exposed as a result of inflammation driven tissue damage and epitope spreading. It is important to note that in autoimmune disease in general not all autoantibodies are pathogenic, and only those targeting cell surface proteins are generally thought to cause clinical manifestations (51). In a similar vein, the anti-myosin autoantibodies observed in ARF (8, 12, 36, 40) could well be the result of tissue damage and cardiomyocyte burst given the intracellular location of myosin within the myocardium (52).

This study has several limitations. The ProtoArrays antigens are expressed in insect cells such that the glycosylation patterns on the extracellular antigens will differ from those that maybe present in human tissue. Similarly, membrane associated antigens may be mis-folded or under-represented on both of the array platforms utilised given the uniform approach required to express and purify such large numbers of antigens in parallel. To overcome this limitation, and expand the antigen space examined in the context of ARF, alternative approaches such as Phage Immunoprecipitation Sequencing [PhiP−Seq (53)] and Rapid Extracellular Antigen Profiling [REAP (54)] of the human proteome could be considered in future studies. Finally, the initial array analysis was based on small patient numbers and it is possible that additional ARF autoantibodies would be detected with larger cohorts. Despite this, the analysis and filtering approach applied to the array data successfully identified three novel ARF autoantigens, each validated in a large patient cohort, supporting the use of high content arrays as a discovery tool.

In conclusion, this study has utilized high content protein array platforms to assess autoantibodies present in ARF in an unbiased fashion. The broad yet heterogenous elevation of autoantibodies in ARF patients support a pathogenesis model in which tissue damage and inflammation leads to a loss of tolerance to endogenous proteins and subsequent epitope spreading. Whilst autoantibodies have diagnostic potential, a panel comprising multiple antigens will likely be needed to capture individual heterogeneity.

## Data Availability Statement

The original contributions presented in the study are included in the article/**Supplementary Material**. Further inquiries can be directed to the corresponding author.

## Ethics Statement

The studies involving human participants were reviewed and approved by Health and Disability ethics committee with the following ethics approval numbers (CEN/12/06/017, 14/NTA/53, 17/NTA/262). Written informed consent to participate in this study was provided by the participants’ or their legal guardian/next of kin.

## Author Contributions

NM, RM, NW, WM, and MB conceived the study. NM, RM, MT, LC, PH-M, JR, WF, DB, MM, PA, and JB performed the experiments and/or acquired the data. RM, NM, MT, and LC analyzed the data. RM and NM wrote the first draft of the manuscript. All authors contributed to the article and approved the submitted version.

## Funding

This work was supported by funding from the Maurice Wilkins Centre, the Heart Foundation of New Zealand (small project grant #1576) and Cure Kids (project grants #3545 and #3585). NM was funded by a Heart Foundation Senior Research Fellowship for part of the study period (#1650). The RF RISK study, from which samples were obtained, was funded by the Heath Research Council of New Zealand (HRC) Rheumatic Fever Research Partnership (Ministry of Health, Te Puni Kōkiri, Cure Kids, Heart Foundation, and HRC).

## Conflict of Interest

The authors declare that the research was conducted in the absence of any commercial or financial relationships that could be construed as a potential conflict of interest.

## References

[1] Wilson NJ Voss L Morreau J Stewart J Lennon D. New Zealand Guidelines for the Diagnosis of Acute Rheumatic Fever: Small Increase in the Incidence of Definite Cases Compared to the American Heart Association Jones Criteria. New Z Med J (2013) 126:50–9. 24045352

[2] Gewitz MH Baltimore RS Tani LY Sable CA Shulman ST Carapetis J. Revision of the Jones Criteria for the Diagnosis of Acute Rheumatic Fever in the Era of Doppler Echocardiography A Scientific Statement From the American Heart Association. Circulation (2015) 131:1806–18. 10.1161/CIR.0000000000000205 25908771

[3] Carapetis JR Steer AC Mulholland EK Weber M. The Global Burden of Group A Streptococcal Diseases. Lancet Infect Dis (2005) 5:685–94. 10.1016/s1473-3099(05)70267-x 16253886

[4] Watkins DA Johnson CO Colquhoun SM Karthikeyan G Beaton A Bukhman G. Global, Regional, and National Burden of Rheumatic Heart Disease, 1990–2015. New Engl J Med (2017) 377:713–22. 10.1056/nejmoa1603693 28834488

[5] Parnaby MG Carapetis JR. Rheumatic Fever in Indigenous Australian Children. J Paediatr Child H (2010) 46:527–33. 10.1111/j.1440-1754.2010.01841.x 20854325

[6] Bennett J Zhang J Leung W Jack S Oliver J Webb R. Rising Ethnic Inequalities in Acute Rheumatic Fever and Rheumatic Heart Disease. Emerg Infect Dis (2021) 27:36–46. 10.3201/eid2701.191791 PMC7774562 33350929

[7] Thomas S Bennett J Jack S Oliver J Purdie G Upton A. Descriptive Analysis of Group A Streptococcus in Skin Swabs and Acute Rheumatic Fever, Auckland, New Zealand, 2010–2016. Lancet Regional Heal - West Pac (2021) 8:100101. 10.1016/j.lanwpc.2021.100101 PMC8315459 34327427

[8] Carapetis JR Beaton A Cunningham MW Guilherme L Karthikeyan G Mayosi BM. Acute Rheumatic Fever and Rheumatic Heart Disease. Nat Rev Dis Primers (2016) 2:15084. 10.1038/nrdp.2015.84 27188830 PMC5810582

[9] Cunningham MW. Streptococcus and Rheumatic Fever. Curr Opin Rheumatol (2012) 24:408–16. 10.1097/bor.0b013e32835461d3 PMC3645882 22617826

[10] Tandon R Sharma M Chandrashekhar Y Kotb M Yacoub MH Narula J. Revisiting the Pathogenesis of Rheumatic Fever and Carditis. Nat Rev Cardiol (2013) 10:171–7. 10.1038/nrcardio.2012.197 23319102

[11] Carapetis JR McDonald M Wilson NJ. Acute Rheumatic Fever. Lancet (2005) 366:155–68. 10.1016/s0140-6736(05)66874-2 16005340

[12] Karthikeyan G Guilherme L. Acute Rheumatic Fever. Lancet (2018) 392:161–74. 10.1016/S0140-6736(18)30999-1 30025809

[13] Rosenberg JM Utz PJ. Protein Microarrays: A New Tool for the Study of Autoantibodies in Immunodeficiency. Front Immunol (2015) 6:138. 10.3389/fimmu.2015.00138 25904912 PMC4387933

[14] Gruber C Patel RS Trachtman R Lepow L Amanat F Krammer F. Mapping Systemic Inflammation and Antibody Responses in Multisystem Inflammatory Syndrome in Children (Mis-C). Cell (2020) 183:1–14. 10.1016/j.cell.2020.09.034 32991843 PMC7489877

[15] Zaenker P Lo J Pearce R Cantwell P Cowell L Lee M. A Diagnostic Autoantibody Signature for Primary Cutaneous Melanoma. Oncotarget (2018) 9:30539–51. 10.18632/oncotarget.25669 PMC6078131 30093967

[16] Chong BF Tseng L Lee T Vasquez R Li QZ Zhang S. Igg and IgM Autoantibody Differences in Discoid and Systemic Lupus Patients. J Invest Dermatol (2012) 132:2770–9. 10.1038/jid.2012.207 PMC3465644 22763789

[17] Ritchie ME Silver J Oshlack A Holmes M Diyagama D Holloway A. Smyth Gk. A Comparison of Background Correction Methods for Two-Colour Microarrays. Bioinformatics (2007) 23:2700–7. 10.1093/bioinformatics/btm412 17720982

[18] Ritchie ME Phipson B Wu D Hu Y Law CW Shi W. Limma Powers Differential Expression Analyses for RNA-Sequencing and Microarray Studies. Nucleic Acids Res (2015) 43:e47–7. 10.1093/nar/gkv007 PMC4402510 25605792

[19] Yang L Wang J Li J Zhang H Guo S Yan M. Identification of Serum Biomarkers for Gastric Cancer Diagnosis Using a Human Proteome Microarray. Mol Cell Proteomics (2016) 15:614–23. 10.1074/mcp.m115.051250 PMC4739676 26598640

[20] Wickham H Averick M Bryan J Chang W McGowan L François R. Welcome to the Tidyverse. J Open Source Softw (2019) 4:1686. 10.21105/joss.01686

[21] Gu Z Eils R Schlesner M. Complex Heatmaps Reveal Patterns and Correlations in Multidimensional Genomic Data. Bioinformatics (2016) 32:2847–9. 10.1093/bioinformatics/btw313 27207943

[22] Bardou P Mariette J Escudié F Djemiel C Klopp C. Jvenn: An Interactive Venn Diagram Viewer. BMC Bioinf (2014) 15:293. 10.1186/1471-2105-15-293 PMC4261873 25176396

[23] Zhou Y Zhou B Pache L Chang M Khodabakhshi AH Tanaseichuk O. Metascape Provides a Biologist-Oriented Resource for the Analysis of Systems-Level Datasets. Nat Commun (2019) 10:1523. 10.1038/s41467-019-09234-6 30944313 PMC6447622

[24] Piñero J Ramírez-Anguita JM Saüch-Pitarch J Ronzano F Centeno E Sanz F. The DisGeNET Knowledge Platform for Disease Genomics: 2019 Update. Nucleic Acids Res (2019) 48:D845–55. 10.1093/nar/gkz1021 PMC7145631 31680165

[25] Uhlén M Fagerberg L Hallström BM Lindskog C Oksvold P Mardinoglu A. Tissue-Based Map of the Human Proteome. Science (2015) 347:1260419. 10.1126/science.1260419 25613900

[26] Binder JX Pletscher-Frankild S Tsafou K Stolte C O’Donoghue SI Schneider R. COMPARTMENTS: Unification and Visualization of Protein Subcellular Localization Evidence. Database (2014) 2014). 10.1093/database/bau012 PMC3935310 24573882

[27] Robin X Turck N Hainard A Tiberti N Lisacek F Sanchez J-C. pROC: An Open-Source Package for R and S+ to Analyze and Compare ROC Curves. BMC Bioinf (2011) 12:77. 10.1186/1471-2105-12-77 PMC3068975 21414208

[28] Atatoa-Carr P Lennon D Wilson N Group NZRFGW. Rheumatic Fever Diagnosis, Management, and Secondary Prevention: A New Zealand Guideline. New Z Med J (2008) 121:59–69. 18392063

[29] McCowan LME Dekker GA Chan E Stewart A Chappell LC Hunter M. Spontaneous Preterm Birth and Small for Gestational Age Infants in Women Who Stop Smoking Early in Pregnancy: Prospective Cohort Study. BMJ Br Med J (2009) 338:b1081. 10.1136/bmj.b1081 19325177 PMC2661373

[30] Baker MG Gurney J Oliver J Moreland NJ Williamson DA Pierse N. Risk Factors for Acute Rheumatic Fever: Literature Review and Protocol for a Case-Control Study in New Zealand. Int J Environ Res Pu (2019) 16:4515. 10.3390/ijerph16224515 PMC6888501 31731673

[31] Salmond C Crampton P King P Waldegrave C. Nzidep: A New Zealand Index of Socioeconomic Deprivation for Individuals. Soc Sci Med (2006) 62:1474–85. 10.1016/j.socscimed.2005.08.008 16154674

[32] Bennett J Moreland NJ Oliver J Crane J Williamson DA Sika-Paotonu D. Understanding Group A Streptococcal Pharyngitis and Skin Infections as Causes of Rheumatic Fever: Protocol for a Prospective Disease Incidence Study. BMC Infect Dis (2019) 19:633. 10.1186/s12879-019-4126-9 31315580 PMC6637506

[33] Slight-Webb S Lu R Ritterhouse LL Munroe ME Maecker HT Fathman CG. Autoantibody-Positive Healthy Individuals Display Unique Immune Profiles That may Regulate Autoimmunity. Arthritis Rheumatol (2016) 68:2492–502. 10.1002/art.39706 PMC5042816 27059145

[34] Prüßmann J Prüßmann W Recke A Rentzsch K Juhl D Henschler R. Co-Occurrence of Autoantibodies in Healthy Blood Donors. Exp Dermatol (2014) 23:519–21. 10.1111/exd.12445 24816528

[35] Neiman M Hellström C Just D Mattsson C Fagerberg L Schuppe-Koistinen I. Individual and Stable Autoantibody Repertoires in Healthy Individuals. Autoimmunity (2019) 52:1–11. 10.1080/08916934.2019.1581774 30835561

[36] Martins TB Hoffman JL Augustine NH Phansalkar AR Fischetti VA Zabriskie JB. Comprehensive Analysis of Antibody Responses to Streptococcal and Tissue Antigens in Patients With Acute Rheumatic Fever. Int Immunol (2008) 20:445–52. 10.1093/intimm/dxn004 18245783

[37] Dinkla K Talay SR Mörgelin M Graham RMA Rohde M Nitsche-Schmitz DP. Crucial Role of the CB3-Region of Collagen IV in PARF-Induced Acute Rheumatic Fever. PloS One (2009) 4:e4666. 10.1371/journal.pone.0004666 19252743 PMC2646144

[38] Pilapitiya DH Harris PW Hanson-Manful P McGregor R Kowalczyk R Raynes JM. Antibody Responses to Collagen Peptides and Streptococcal Collagen-Like 1 Proteins in Acute Rheumatic Fever Patients. Pathog Dis (2021) 79. 10.1093/femspd/ftab033 PMC8600009 34185083

[39] Jones KF Whitehead SS Cunningham MW Fischetti VA. Reactivity of Rheumatic Fever and Scarlet Fever Patients’ Sera With Group A Streptococcal M Protein, Cardiac Myosin, and Cardiac Tropomyosin: A Retrospective Study. Infect Immun (2000) 68:7132–6. 10.1128/iai.68.12.7132-7136.2000 PMC97825 11083840

[40] Towers RJ Bolm M Currie BJ Chhatwal GS Fagan PK. Autoantigens Identified by Screening a Human Heart Cdna Library With Acute Rheumatic Fever Sera. Ann N Y Acad Sci (2008) 1173:83–91. 10.1111/j.1749-6632.2009.04653.x 19758136

[41] Rhee I Veillette A. Protein Tyrosine Phosphatases in Lymphocyte Activation and Autoimmunity. Nat Immunol (2012) 13:439–47. 10.1038/ni.2246 22513334

[42] Fairclough RJ Wood MJ Davies KE. Therapy for Duchenne Muscular Dystrophy: Renewed Optimism From Genetic Approaches. Nat Rev Genet (2013) 14:373–8. 10.1038/nrg3460 23609411

[43] Matteo RG Moravec CS. Immunolocalization of Annexins IV, V and VI in the Failing and Non-Failing Human Heart. Cardiovasc Res (2000) 45:961–70. 10.1016/s0008-6363(99)00409-5 10728422

[44] Campbell KA Minashima T Zhang Y Hadley S Lee YJ Giovinazzo J. Annexin A6 Interacts With p65 and Stimulates Nf-κb Activity and Catabolic Events in Articular Chondrocytes. Arthritis Rheumatism (2013) 65:3120–9. 10.1002/art.38182 24022118

[45] Youden WJ. Index for Rating Diagnostic Tests. Cancer (1950) 3:32–5. 10.1002/1097-0142(1950)3:1<32::aid-cncr2820030106>3.0.co;2-3 15405679

[46] Schett G Tanaka Y Isaacs JD. Why Remission is Not Enough: Underlying Disease Mechanisms in RA That Prevent Cure. Nat Rev Rheumatol (2021) 17:135–44. 10.1038/s41584-020-00543-5 33303993

[47] Raynes JM Frost HR Williamson DA Young PG Baker EN Steemson JD. Serological Evidence of Immune Priming by Group A Streptococci in Patients With Acute Rheumatic Fever. Front Microbiol (2016) 7:1119. 10.3389/fmicb.2016.01119 27499748 PMC4957554

[48] Zabriskie JB. Mimetic Relationships Between Group A Streptococci And Mammalian Tissues. Adv Immunol (1967) 7:147–88. 10.1016/s0065-2776(08)60128-5 4868522

[49] Lorenz N Ho TKC McGregor R Davies MR Williamson DA Gurney JK. Serological Profiling of Group A Streptococcus Infections in Acute Rheumatic Fever. Clin Infect Dis (2021), ciab180. 10.1093/cid/ciab180 33639619

[50] Chung AW Ho TK Hanson-Manful P Tritscheller S Raynes JM Whitcombe AL. Systems Immunology Reveals a Linked IgG3–C4 Response in Patients With Acute Rheumatic Fever. Immunol Cell Biol (2019) 98:12–21. 10.1111/imcb.12298 31742781

[51] Naparstek Y Plotz PH. The Role of Autoantibodies in Autoimmune Disease. Annu Rev Immunol (1993) 11:79–104. 10.1146/annurev.iy.11.040193.000455 8476578

[52] Root-Bernstein R. Rethinking Molecular Mimicry in Rheumatic Heart Disease and Autoimmune Myocarditis: Laminin, Collagen IV, CAR, and B1AR as Initial Targets of Disease. Front Pediatr (2014) 2:85. 10.3389/fped.2014.00085 25191648 PMC4137453

[53] Mohan D Wansley DL Sie BM Noon MS Baer AN Laserson U. PhIP-Seq Characterization of Serum Antibodies Using Oligonucleotide-Encoded Peptidomes. Nat Protoc (2018) 13:1958–78. 10.1038/s41596-018-0025-6 PMC6568263 30190553

[54] Wang EY Dai Y Rosen CE Schmitt MM Dong MX Ferré EMN. Reap: A Platform to Identify Autoantibodies That Target the Human Exoproteome. Biorxiv (2021). 10.1101/2021.02.11.430703

