# Supplementary material for: Mapping Autoantibodies in Children With Acute Rheumatic Fever
Source: Front Immunol. 2021 Jul 15;12:702877. doi: 10.3389/fimmu.2021.702877 (PMC8320770; doi:10.3389/fimmu.2021.702877)

Supplementary Material

Supplementary Table. Demographic Characteristics of the Study Participants

		**Acute Rheumatic Fever**	**Healthy Controls**	**GAS Pharyngitis***
**Total (n)**		79	85	39
**Age (years)**	Median	11	11	9
	Range	4-18	6-17	5-14
**Sex**	Male (%)	54 (69)	55 (65)	21 (54)
	Feale (%)	24 (31)	30 (35)	18 (46)
**Ethnicity**	Māori (%)	27 (34)	34 (40)	9 (23)
	Pacific (%)	52 (66)	51 (60)	13 (33)
	Other (%)	0 (0)	0 (0)	17 (44)

*Serological and throat-swab confirmed GAS infection.

Supplementary Figure 1. Overall antibody binding intensities against human proteins in ARF patients (blue, n=3) pharyngitis controls (dark red, n=3) and healthy controls (light red, n=3). Bars indicate mean and 99% confidence intervals.

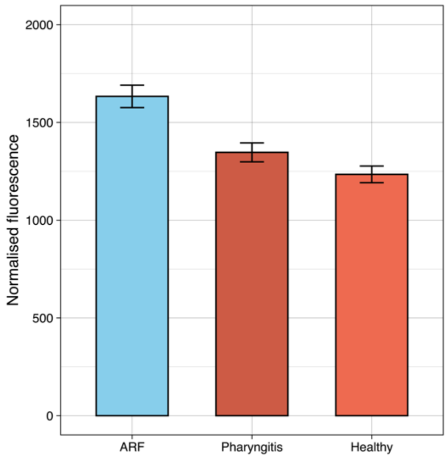

Supplementary Figure 2. Tropomyosin sequence alignment. **(A)** Mulitple sequence alignment of cardiac tropomyosin (TPM1), TPM2 and TPM3 amino acid sequences using Blosum62 Geneious alignment within Geneious (Build 2021-03-12 13:25). Identity row colours; light green = 100% identity, dark green = at least 30% and under 100% identity. **(B)** Table showing percentage identity from alignment carried out in part (A).

A

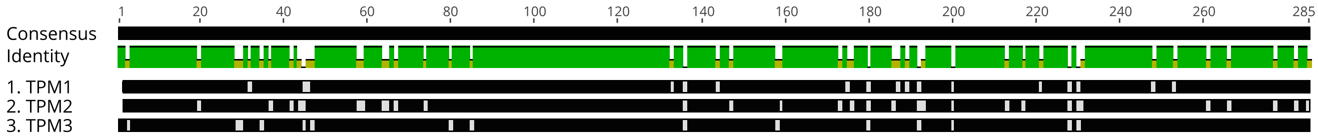

B

	TPM1	TPM2	TPM3
**TPM1**		85.563	91.197
**TPM2**	85.563		86.268
**TPM3**	91.197	86.268	

Supplementary methods

Two-dimensional Gel Electrophoresis (2-DE)

Whole tissue heart lysate (60ug/strip) from a healthy human donor (Novus Biologicals, Colorado, USA) was applied to non-linear immobilized pH gradient strips (11cm, pH3-10, Bio-Rad, California, USA) and strips were rehydrated overnight at room temperature. Isoelectric focusing (IEF) was performed using Multiphor II (GE Healthcare, Illinois, USA) for 30 h. After IEF, strips were embedded in 10% SDS-PAGE gel and the gels were run for 3h on a Protean II XL cell (Bio-Rad). Following electrophoresis, one gel was stained with colloidal Coomassie for visualization of separated proteins. The remaining two gels were transferred to PVDF membranes and blocked in 5% Milk/PBS for 12 h at 4°C. Following washing in PBS, blots were incubated with human sera (ARF or pooled healthy controls) diluted 1:250 in 5% Milk/PBS supplemented with 0.1% Tween-20 (5%MT) for 1.5 h at room temperature. After washing with PBS-T, the membranes were incubated with horseradish peroxidase (HRP) labelled goat anti-human IgG (abcam, Cambridge, UK; 1:5,000 dilution) for 1.5 h and bound antibodies were visualized using enhanced luminol-based chemiluminescent (ECL) (ThermoFisher Scientific) on a ChemiDoc system (Bio-Rad).

Mass spectrometry

Spots of interest were ascertained by comparing blots probed with either ARF or healthy donor sera, with those only detected on the ARF blot selected for further analysis. These spots were cut out of the 2-DE, subjected to controlled proteolysis with trypsin (Promega, Madison, WY, USA) and identified using a TripleTOF 6600 LC-MS/MS system (Sciex, Massachusetts, USA) and Protein Pilot analysis software (Sciex). Two adjacent spots identified Annexin A6, with total scores of 60.82 and 77.25, respectively (Supplementary Methods Figure). The presence of Annexin A6 in adjacent spots likely indicates different isoforms or glycoforms.

Supplementary Methods Figure

Two-dimensional Gel Electrophoresis (2-DE) of heart tissue. Spots detected with ARF sera and not healthy control sera, which were subsequently identified as Annexin A6 are indicated.

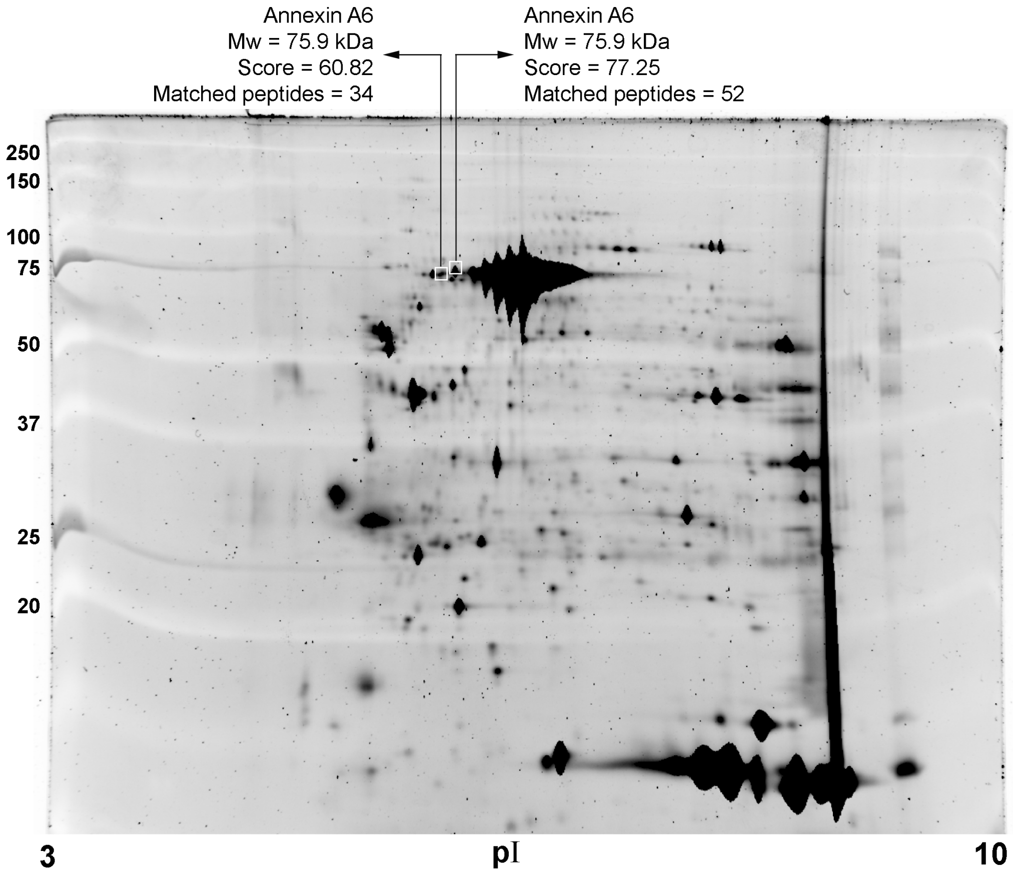

Supplement: Supplementary file 1 [file DataSheet_1.pdf]
